# Advancing Health Equity for Asian Americans, Native Hawaiians, and Pacific Islanders

**DOI:** 10.1089/heq.2022.0075

**Published:** 2022-06-02

**Authors:** Krystal Ka'ai, Rebecca Lee, Victoria Chau, Lanlan Xu

**Affiliations:** Department of Health and Human Services, White House Initiative on Asian Americans, Native Hawaiians, and Pacific Islanders (WHIAANHPI), Washington, District of Columbia, USA.

**Keywords:** health disparities, minority health, racial minority

## Abstract

This article discusses the urgency of focusing on health disparities for the Asian American, Native Hawaiian, and Pacific Islander (AA and NHPI) communities and lays out three key policy priorities for the White House Initiative on Asian Americans, Native Hawaiians, and Pacific Islanders to advance health equity for the AA and NHPI communities: Anti-Asian hate and violence, data disaggregation, and language access.

## Background

May is Asian American, Native Hawaiian, and Pacific Islander (AA and NHPI) Heritage Month. While we celebrate AA and NHPIs, exacerbated long-standing inequities have contributed to this population being disproportionately impacted by the COVID-19 pandemic. New studies reveal AAs experience significantly higher excess all-cause mortality (3.1 times higher), case fatality rates (as high as 53% higher), and percentage of deaths attributed to COVID-19 (2.1 times higher) compared with non-Hispanic Whites.^1^ In early 2020, NHPIs had the highest COVID-19 case rates within specific states and counties compared with all racial and ethnic groups,^2^ and of 20 states that reported fatality rates for NHPIs, 18 of these states had NHPIs representing the highest per capita fatality rate.^3^ Concurrently, violence and racism plagued AA communities—increasing by 339% last year^4^—and aggravated AAs' physical and mental health problems. These dual crises highlight the urgency of addressing health equity issues for AA and NHPI communities.

AA and NHPIs are among the fastest growing racial/ethnic groups in the United States, representing ∼24.5 million people in 2019.^5^ Comprising >50 distinct ethnic groups that speak >100 languages and dialects, with differing immigration statuses, socioeconomic standings, and geographic backgrounds, AA and NHPIs are not a monolithic group.^6^ However, these diverse communities are often overlooked or misrepresented in mainstream narratives concerning health disparities. Methodology issues with data collection have excluded AA and NHPI populations from decades of critical research, while the pandemic exacerbated these issues and amplified the need for improvement.

## White House Initiative on Asian Americans, Native Hawaiians, and Pacific Islanders

President Joe Biden established the White House Initiative on Asian Americans, Native Hawaiians, and Pacific Islanders (WHIAANHPI) within the U.S. Department of Health and Human Services in May 2021 through Executive Order (EO) 14031: Advancing Equity, Justice, and Opportunity for Asian Americans, Native Hawaiians, and Pacific Islanders.^7^

Alongside 14 policy goals, WHIAANHPI works to address key issues that impact the health and well-being of AA and NHPIs, including anti-AA and NHPI hate and bias; data disaggregation; and language access. These three priority areas cut across multiple issues and intersect to affect health equity, which is outlined in EO 14031 as to improve health outcomes, eliminate health disparities, and expand access to quality, affordable, and culturally competent medical and mental health care services for AA and NHPI individuals and communities.

In this article, we focus on these three priority areas' impacts on health equity, while acknowledging there are additional intersections not discussed here.

### Anti-Asian hate/violence

Racism against AA and NHPIs is not new, but the blatant assaults, fueled by xenophobic rhetoric that associated AAs with the severe acute respiratory syndrome coronavirus 2 (SARS-CoV-2) virus, intensified during the COVID-19 pandemic. From March 19, 2020 to December 31, 2021, a total of 10,905 hate incidents against AA and NHPIs were reported to Stop AAPI Hate.^8^

Anti-AA and NHPI sentiments also pervaded health care and business. Nationally, the number of Asian-owned businesses fell by 20% due to the pandemic and associated discrimination.^9^ In health care, where AA and NHPIs account for 8.5% of the field, refusals of care and verbal attacks became common.^10^

The public brutal attacks of AAs, coupled with discrimination in health care and business, negatively impacted the mental health of AA communities. Addressing anti-Asian hate is imperative to healing AA communities.^11^

Since being in office, President Biden has called on federal government to address anti-Asian hate and violence, issuing a Presidential Memorandum and signing the COVID-19 Hate Crimes Act.^12^ WHIAANHPI is raising awareness of hate crimes and disseminating federal resources for AA and NHPIs.^13^

### Data disaggregation and language access

Lack of disaggregated data and language access for AA and NHPIs have consistently stifled health equity. Historically, AA and NHPIs have been left out of data collection and reporting, and when included, the data are seldom disaggregated. This is especially harmful for NHPI and certain Southeast Asian communities, who face significant disparities that are often masked when AA and NHPI data are aggregated.

Research showing NHPI communities having the highest COVID-19 death rates in the United States raised the alarm about the disproportionate impact of the virus.^3^ Documenting these health disparities with disaggregated data are vital to reaching health equity. Providing translated materials and interpretive services is an essential piece to ensuring linguistic differences do not prevent access to critical resources.

Several presidential actions direct the federal government to address these systemic issues. President Biden's EO 13994 and 13995 charge the federal government to implement a data-driven and equitable response to COVID-19; EO 13985 and EO 14031 focus on advancing equity for racial/ethnic minorities, including AA and NHPIs; and President Bill Clinton's EO 13166 centers on improving language access for limited English proficient (LEP) populations. WHIAANHPI is committed to working with agencies across the federal government to improve data collection and reporting as well as to provide culturally and linguistically appropriate services to AA and NHPIs.^14–18^

## Conclusion

Tackling long-standing health disparities that COVID-19 exacerbated requires an intersectional holistic approach that honors diversity. AA and NHPIs are often seen as a monolithic group, left out of health equity discourse, and/or simply made invisible. WHIAANHPI and the Biden–Harris Administration are committed to eliminating anti-AA and NHPI hate and discrimination, collecting and providing disaggregated AA and NHPI data, and improving language accessibility for LEP communities. This Administration's record of addressing health equity for AA and NHPIs includes expanding health care access to AA and NHPIs through Marketplace tax credits from the passing of the American Rescue Plan Act.^19^

We must take this opportunity to act now, not just for the sake of AA and NHPIs, but for the health of our nation. WHIAANHPI looks forward to working with government agencies, civic society, and community members to advance equity, justice, and opportunity for AA and NHPI communities.

## Abbreviations Used

AA and NHPI: Asian American, Native Hawaiian, and Pacific Islander
EO: Executive Order
LEP: limited English proficient
WHIAANHPI: White House Initiative on Asian Americans, Native Hawaiians, and Pacific Islanders
